# Impact of Nirsevimab on bronchiolities in pediatric primary care in Lazio Region: an observational study

**DOI:** 10.1186/s13052-026-02221-w

**Published:** 2026-03-13

**Authors:** Daniele Petrone, Ilaria Sani, Valentina De Vittori, Silvana Brenna, Pietro Luigi Rotili, Maria Teresa Fonte

**Affiliations:** 1 Department of Infectious Diseases, Istituto Superiore di Sanità, Viale Regina Elena, 299, 00161 Rome, Italy; 2 Department of Statistics, Sapienza University of Rome, Rome, Italy; 3 Primary Care Pediatrician, FIMP Roma, Rome, Italy

**Keywords:** Nirsevimab, Immunization, RSV, Prevention

## Abstract

**Background:**

Bronchiolitis is the leading cause of lower respiratory tract infections in infants, with respiratory syncytial virus (RSV) as the primary pathogen. Nirsevimab, a long-acting monoclonal antibody, was introduced in Europe in late 2022 for RSV prophylaxis in all infants. In Italy, the 2024–2025 immunization campaign faced regional disparities in implementation. This study aimed to evaluate the impact of Nirsevimab in reducing bronchiolitis diagnoses and hospitalizations in infants during their first RSV season in the Lazio Region.

**Methods:**

We conducted a retrospective cohort study using data from 29 primary care pediatricians in Lazio. Infants born between August 17, 2024, and March 31, 2025, were included. Bronchiolitis diagnoses were clinically defined, and immunization status was obtained from the regional vaccination registry. We excluded premature infants (< 33 weeks) and those diagnosed before the immunization campaign began on December 9, 2024. To adjust for confounding, we applied inverse probability weighting (IPW) based on a propensity score including demographic and clinical covariates. A negative binomial mixed-effects model was used to estimate incidence rate ratios (IRRs) for bronchiolitis and hospitalizations.

**Results:**

Among 818 eligible infants, 613 (74.9%) were immunized. A total of 58 bronchiolitis cases were recorded (7.1%), with 6.5% in the immunized group and 8.8% in the non-immunized group. Crude analysis showed a 25.7% risk reduction, while IPW-adjusted analysis indicated a 50.4% reduction (95% CI: 44.4%–55.7%). Hospitalizations occurred in 2.0% of infants, with adjusted analysis showing a 49.1% reduction in hospitalization risk among immunized infants. The immunization campaign’s late start likely limited its full impact, as 40 early-season cases were excluded.

**Conclusions:**

Nirsevimab immunization significantly reduced the risk of bronchiolitis and related hospitalizations in a real-world primary care setting. This result aligns with previous studies, though it may be underestimated due to the delayed campaign start and inclusion of all-cause bronchiolitis. These findings support early and widespread implementation of Nirsevimab to optimize protection and reduce RSV burden in infants.

## Background

Bronchiolitis is the most common lower respiratory tract infection among infants and young children. Respiratory syncytial virus (RSV) is the leading causative agent of bronchiolitis and is estimated to be responsible for 33.1 million cases of lower respiratory tract infection in children younger than 5 years annually, with 3.2 million hospitalizations and more than 100,000 deaths worldwide each year [1, 2].

Most deaths occur in low-income and developing countries. However, even in countries with higher socio-economic development, RSV represents an important healthcare challenge, which requires a reorganization to admit children requiring hospitalization and intensive care for severe bronchiolitis, especially in winter months when RSV typically circulates [3].

Bronchiolitis under 12 months of age is associated with an increased risk of recurrent wheezing in the following years. The question remains whether respiratory infection at a young age itself predisposes children to asthma through damage or alteration of lung function, or whether children with severe bronchiolitis might have individual risk factors (such as altered immune response or airway function) that predisposes them to severe bronchiolitis and recurrent wheezing [4].

Until 2023, Palivizumab was the only approved agent for RSV prophylaxis, and its indications were limited to high-risk infants. Nirsevimab, approved by the European Medical Agency in November 2022 and by the Food and Drug Administration in July 2023, is a monoclonal antibody against both A and B type RSV that has an enhanced neutralizing activity and extended half-life in vivo, which enable its implementation in the general population [5]. Double-blind, randomized, placebo-controlled trial among healthy preterm and full-term infants have shown that Nirsevimab had an efficacy of 74.5% in reducing the risk of RSV-associated lower respiratory tract infection and of 62.1% in decreasing RSV-associated hospitalizations [6]. Another recent randomized, controlled, phase 3b study conducted in France, Germany, and the UK (HARMONIE), showed that Nirsevimab offers in infants consistent and sustained protection against hospitalization due to RSV-associated lower respiratory tract infection for at least 5 months [7, 8].

Nirsevimab immunization started in the autumn of 2023 in few countries, the post licensure real-world effectiveness of Nirsevimab in the prevention of RSV-associated bronchiolitis still needs to be studied and is much debated. Few post-license studies have been published on the effectiveness of Nirsevimab, most of them focus on hospitalized patients.

In Italy, according to the Resolution of State-Regions Conference of 17th October 2024, the immunization campaign with Nirsevimab was expected to start on 1st November 2024 in all newborns, extending coverage to children born in the previous 100 days and to children with fragile conditions [9]. Unfortunately, due to low doses availability and some economic issues, the immunization campaign involved different infant groups in each Italian region. Some of them offered the immunization campaign only to children born during the RSV epidemic season, other regions proposed Nirsevimab also to children born before RSV season. Moreover, the immunization starting date was different in every Italian region, from November 2024 to January 2025 [10]. This discrepancy generated perceptions of inequality, access issues, and criticism toward initial immunization decisions [11].

In Lazio Region the immunization campaign began on 25th November 2024 for newborns, according to regional deliberation and was concretely offered from 9th December to all children born from 17th of August 2024 [12, 13].

The objective of the study is to assess Nirsevimab’s impact on all-cause bronchiolitis diagnosis in a primary care setting in infants born between 17 August 2024 and 31 March 2025 during their first epidemic season in the Lazio Region. Moreover, we analyzed in our population the hospitalization rate and estimated the impact of Nirsevimab on this outcome.

## Materials and methods

### Study design and data sources

We conducted a retrospective cohort study using routinely collected electronic medical records from 29 family pediatricians of primary care (PPCs). All PPCs involved in the study worked in Lazio Region, Italy and they were enrolled on a voluntary basis. In this Region, there are currently 727 PPCs, providing care to an average of 910 patients aged 0 to 16. PPCs work in individual medical studies or associated with other physicians and provide primary medical care, health assessments, prevention activities, and vaccinations. Among these 727, only 29 PPCs, representative of many areas of the region, participated voluntarily in this pilot study. The PPCs involved in the study collected demographic and clinical information of the selected population, including any bronchiolitis event and any hospitalization due to bronchiolitis. The diagnosis of bronchiolitis was clinically defined, as the first episode of viral disease occurring in children under 12 months of age during the autumn-winter epidemic season, characterized at onset by rhinorrhea and upper respiratory tract symptoms, associated with crackles and/or wheezing, use of accessory muscles or lower chest wall retractions, low O2 saturation levels, high respiratory rate relative to age, skin color changes, nasal flaring, fever or feeding difficulties [14].

Nirsevimab exposure was communicated to the PPCs from the Regional Vaccination Register (AVR), an electronic database of all vaccinations performed for a single patient. Each PPC used a spreadsheet previously created and approved by all participants, so that data were collected in a uniform manner.

### Population under study and outcomes

We initially collected data from all the infants born from 01 April 2024 to 31 March 2025 to include the entire at-risk population under 1 year of age during the 2024–2025 RSV season. Then we excluded from the analysis children who were born before 17 August 2024 who were not targeted by the immunization campaign according to the Lazio regional guidelines [13]. We excluded premature infants (< 33 weeks) because, being at high risk, they received a first dose of Palivizumab before the Nirsevimab immunization start date. As prematurity is defined as gestational age < 37 weeks, moderate to late preterm infants (between 33 and 37 weeks) were eligible for and included in the study.

In our population those who received Nirsevimab had a single intramuscular injection at the dose of 50 mg for infants weighing less than 5 kg and 100 mg for those weighing at least 5 kg. No serious adverse events were observed after Nirsevimab administration, particularly no adverse reaction required medical attention.

We recorded all bronchiolitis events that occurred from 01 November 2024 to 31 March 2025, but we excluded from the statistical analysis children who had a bronchiolitis event before the real immunization start date, 09 December 2024. All data was communicated by each PPC in the first two weeks of May 2025 to avoid possible delays in reporting and to allow all necessary information to be obtained.

### Statistical analysis

We described the main characteristics of infants and the frequency of the outcome of interest using counts and percentages. We evaluated the association between the dichotomous variable related to bronchiolitis event and immunization using the unadjusted and adjusted method. In the unadjusted method, to estimate the reduction of the risk associated with immunization, we used the formula ((1-RR)*100), where RR is the relative risk obtained as ratio of the incidence of cases among immunized and not immunized infants. In the adjusted method, we evaluated the association between the dichotomous dependent variable (bronchiolitis event) and having immunization. To consider possible imbalance between the immunized and not immunized groups in the observed covariates, we applied inverse probability weighting (IPW) based on the propensity score (PS). The PS represented the probability of receiving immunization, estimated through a logistic regression model adjusted by the following covariates: sex (Male/Female), month of birth (Aug-Sep/Oct/Nov/Dec/Jan/Feb/Mar), congenital heart disease (Yes/No), immune system disorders (Yes/No), neurological system disorders (Yes/No), mother’s influenza vaccination during pregnancy (Yes/No), type of breastfeeding (Exclusive/Artificial/Mixed), presence of siblings (Yes/No), number of smoking parents (0/1/2), number of atopic parents (0/1/2), atopy (Yes/No), daycare attendance (Yes/No), having received influenza vaccine (Yes/No). We then defined the weights as 1/PS for immunized subjects and 1/(1 − PS) for not immunized subjects.

Since immunization is a time-dependent variable, before the calculation of the weights, we split the follow-up time for each individual into two observations. The first considers the time elapsed between the start of follow-up (the latest birth date or beginning of the immunization campaign) and the date of immunization. The second observation considers the time from immunization and the end of follow-up (defined as the earliest bronchiolitis diagnosis or study end date). For infants who did not receive immunization, follow-up was represented by a single interval, from start to end of follow-up. Finally, we fitted a Negative Binomial mixed-effects model to estimate incidence rate ratios (IRRs). The model included the number of person-days as an offset and the PPC as a random effect to taking into account possible variability in diagnostic practices. We applied the same model to assess the effect of Nirsevimab on hospital admissions for bronchiolitis, using hospitalization as the outcome and the admission date as the event time. To verify the covariates balance after IPW, we used the Standardized Mean Difference (SMD), assuming a satisfactory balance where SMD values were less than 0.1. Estimates from the Negative Binomial Models are presented with their 95% confidence interval (95%CI). Missing data were handled using multiple imputation via predictive mean matching (PMM), generating 5 imputed datasets. Final estimates were combined using Rubin’s rules to account for imputation uncertainty [15]. The reduction of the risk associated with immunization was obtained with the formula ((1 − IRR) x 100). All the analyses were carried out with RStudio 2024.04.2 under R 4.4.1 [16].

## Results

**Fig. 1:**
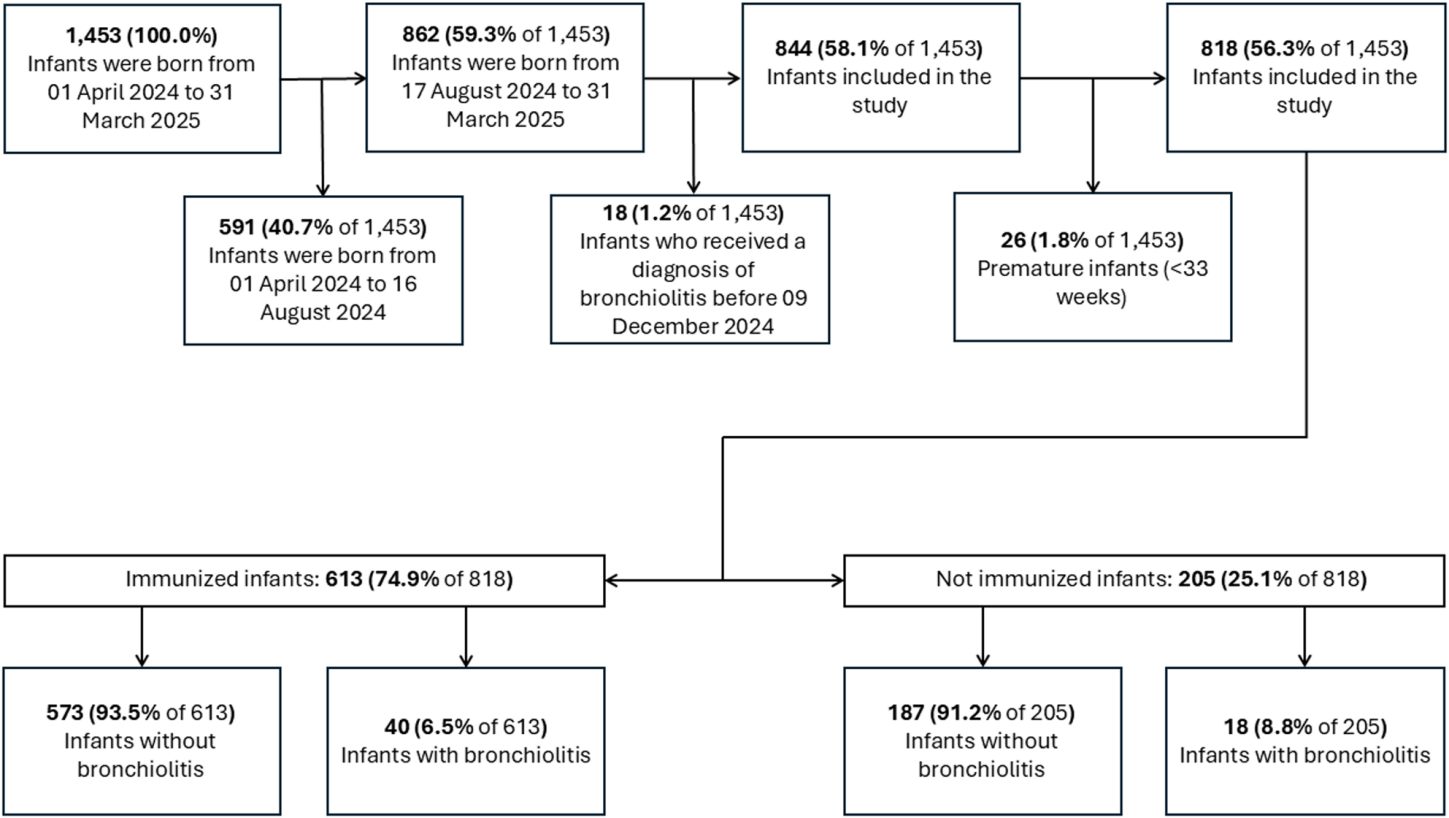
Flowchart of the infants excluded and included from/in the study (study population)

The 29 PPCs involved in the study collected data of 1453 infants who were born from 01 April 2024 to 31 March 2025. Of them, 862 (59.3%, 862/1453) were born between 17 August 2024 and 31 March 2025 and of them 18 (1.2%, 18/1453) received a diagnosis of bronchiolitis between 01 November 2024 and 09 December 2024 and therefore were ruled out from the study. Finally, we excluded 26 infants (1.8%, 18/1453) who were born prematurely. Thus, in our study we included 818 (56.3%, 818/1453) infants (Fig. 1); 613 (74.9%, 613/818) of them received immunization and 205 did not (25.1%, 205/818). Among all subjects, there were 58 (7.1%, 58/818) bronchiolitis events, 18 of them happened in the non-immunized group amounting to 8.8% (18/205) among all the no-immunized infants and 40 of them were registered in the immunized group amounting to 6.5% (40/613) among all the immunized infants.

**Fig. 2:**
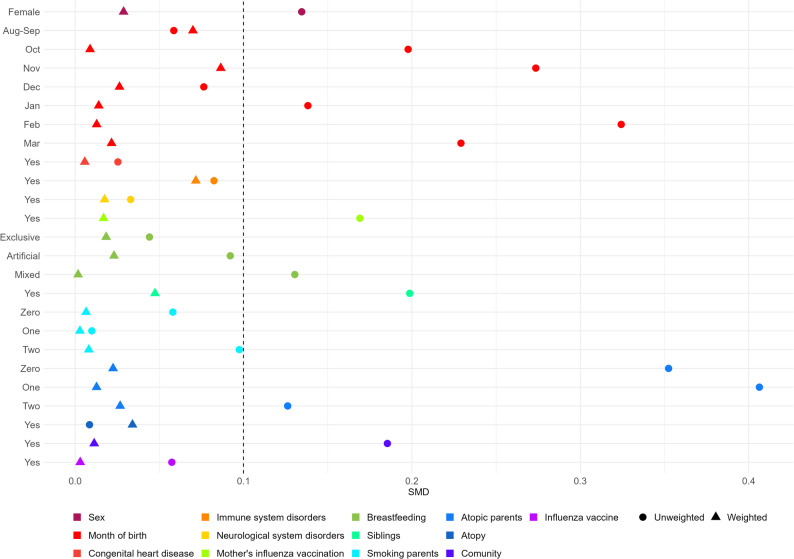
Balance of the covariate used in the models before and after observation weighting with IPW

From the descriptive analysis, no differences emerge between those who have been immunized and those who have not (Table 1). However, the SMD indicates that several baseline characteristics between immunized and not immunized infants appeared unbalanced, suggesting that simple unadjusted comparisons could be influenced by confounding factors. To address this limitation, we applied inverse probability weighting (IPW). After the application of it, the distribution of the main covariates was adequately balanced, reaching the predefined threshold for standardized mean difference (SMD < 0.1) (Fig. 2), which indicates that differences between groups were minimized, and the two populations became more comparable.

**Table 1:** Individual characteristics of infants included in the study before imputation of the missing data

		Immunized infants 613 (74.9%)	Not immunized infants 205 (25.1%)	Total 818 (100.0%)
Bronchiolitis	Yes	40 (6.5%)	18 (8.8%)	58 (7.1%)
	No	573 (93.5%)	187 (91.2%)	760 (92.9%)
Sex	Male	284 (46.3%)	108 (52.7%)	392 (47.9%)
	Female	329 (53.7%)	97 (47.3%)	426 (52.1%)
Congenital Heart Disease	Yes	18 (2.9%)	4 (2%)	22 (2.7%)
	No	592 (96.6%)	195 (95.1%)	787 (96.2%)
	Unknown	3 (0.5%)	6 (2.9%)	9 (1.1%)
Bronchodysplasia	Yes	2 (0.3%)	0 (0%)	2 (0.2%)
	No	609 (99.3%)	198 (96.6%)	807 (98.7%)
	Unknown	2 (0.3%)	7 (3.4%)	9 (1.1%)
Immune System Disorders	Yes	1 (0.2%)	0 (0%)	1 (0.1%)
	No	610 (99.5%)	198 (96.6%)	808 (98.8%)
	Unknown	2 (0.3%)	7 (3.4%)	9 (1.1%)
Neurological System Disorders	Yes	4 (0.7%)	0 (0%)	4 (0.5%)
	No	606 (98.9%)	199 (97.1%)	805 (98.4%)
	Unknown	3 (0.5%)	6 (2.9%)	9 (1.1%)
Mother’s Influenza Vaccination During Pregnancy	Yes	164 (26.8%)	38 (18.5%)	202 (24.7%)
	No	407 (66.4%)	120 (58.5%)	527 (64.4%)
	Unknown	42 (6.9%)	47 (22.9%)	89 (10.9%)
Type of Breastfeeding	Exclusive	351 (57.3%)	100 (48.8%)	451 (55.1%)
	Artificial	93 (15.2%)	38 (18.5%)	131 (16%)
	Mixed	166 (27.1%)	45 (22%)	211 (25.8%)
	Unknown	3 (0.5%)	22 (10.7%)	25 (3.1%)
Presence of Siblings	Yes	260 (42.4%)	91 (44.4%)	351 (42.9%)
	No	350 (57.1%)	96 (46.8%)	446 (54.5%)
	Unknown	3 (0.5%)	18 (8.8%)	21 (2.6%)
Number of Smoking Parents	0	402 (65.6%)	106 (51.7%)	508 (62.1%)
	1	143 (23.3%)	43 (21%)	186 (22.7%)
	2	24 (3.9%)	11 (5.4%)	35 (4.3%)
	Unknown	44 (7.2%)	45 (22%)	89 (10.9%)
Number of Atopic Parents	0	431 (70.3%)	110 (53.7%)	541 (66.1%)
	1	136 (22.2%)	49 (23.9%)	185 (22.6%)
	2	25 (4.1%)	4 (2%)	29 (3.5%)
	Unknown	21 (3.4%)	42 (20.5%)	63 (7.7%)
Atopy	Yes	40 (6.5%)	14 (6.8%)	54 (6.6%)
	No	469 (76.5%)	106 (51.7%)	575 (70.3%)
	Unknown	104 (17%)	85 (41.5%)	189 (23.1%)
Daycare Attendance	Yes	14 (2.3%)	5 (2.4%)	19 (2.3%)
	No	595 (97.1%)	178 (86.8%)	773 (94.5%)
	Unknown	4 (0.7%)	22 (10.7%)	26 (3.2%)

This adjustment had an important impact on the effect estimates. In the crude, unadjusted analysis, immunization with Nirsevimab was associated with a risk reduction of 25.7% (95%CI: -32.5% − 58.3%) (RR: 0.743, (40/613)/(18/205)) in bronchiolitis cases. When the same analysis was repeated using the IPW-adjusted sample, the reduction in risk increased to 50.4% (95%CI: 44.4% − 55.7%), suggesting that the protective effect of Nirsevimab may have been underestimated in the raw data due to imbalances between groups.

During the study period, 16 infants (2.0% of all participants, 16/818) required hospitalization 27.6% (16/58) of those who had bronchiolitis. Of these, 5 (2.4% of not immunized infants, 5/205) had not received immunization, while 11 had (1.8% of immunized infants, 11/613). Moreover, 14 infants were tested for multiple pathogens, and in 10 cases, RSV was detected either alone or alongside other pathogens; in 5 of these, it was detected as a single pathogen, while in the remaining 5 cases, RSV was part of a co-infection with another virus (Table 2). The unadjusted analysis based on hospital admissions due to bronchiolitis showed a 26.4% (95%CI: -114.3% − 74.7%) reduction in risk (R: 0.736, (11/613)/(5/205)) associated with the immunization. The adjusted method showed an increased reduction in risk to 49.1% (95%CI: 43.0% − 54.5%).

**Table 2:** Distribution of viruses detected by swab during hospitalization and information about immunization received

	RSV	Rhinovirus	Enterovirus	HKUI	Flu	Adenoviruses	HMPV	Unknown	IMMUNIZATION
Infant 1	x	x				x			Yes
Infant 2	x			x					Yes
Infant 3	x								Yes
Infant 4	x								No
Infant 5								x	Yes
Infant 6								x	Yes
Infant 7	x								No
Infant 8					x				No
Infant 9		x							Yes
Infant 10	x								Yes
Infant 11	x	x							Yes
Infant 12	x	x							No
Infant 13	x			x					Yes
Infant 14		x		x			x		Yes
Infant 15		x	x						Yes
Infant 16	x								No

* *HKUI*: Human coronavirus HKU1

**HMPV: Human metapneumovirus

## Discussion

This retrospective cohort study, based on data collected by PPCs, aimed to evaluate the impact of Nirsevimab on the prevention of all-cause bronchiolitis in a primary care outpatient setting. While most studies available in literature have focused on severe outcomes such as hospital admissions and complications requiring intensive care, there are still limited data regarding the protective effect of this intervention in the community and in a primary care setting.

Several studies have documented the protective effect of Nirsevimab in different clinical contexts.

An interesting study has been conducted in Tuscany region (Italy), providing a cost–benefit analysis about three possible Nirsevimab immunization strategies compared against the prophylaxis practices in place at the time of the study, which included the use of Palivizumab in eligible infants. The impact of the prophylaxis programs was measured using hospitalizations and severe hospitalizations for RSV acute lower respiratory infections as health outcomes. The results show that universal prophylaxis strategies with Nirsevimab, targeting all infants during their first RSV epidemic season, substantially reduce hospitalization burdens without increasing economic pressure on the healthcare system. Although alternative strategies are more cost-effective, they prevent fewer hospitalizations, emphasizing the public health value of broader prophylaxis approaches [17].

A prospective observational cohort study conducted in Valle d’Aosta, the first region to propose prophylaxis with Nirsevimab, shows that the risk of hospitalization for RSV bronchiolitis in 2023–2024 epidemic season was 3.2%, contrasting with the 7% prevalence observed in the 2022–2023 epidemic season. After the start of the prophylaxis campaign, the risk of hospitalization was 8.3% among infants who did not adhere to the prophylaxis, while no child in the sample of those treated (*p* < 0.001) was hospitalized for bronchiolitis [18].

Another retrospective multicenter study compared RSV seasons before and after universal Nirsevimab prophylaxis implementation, including all live births from 5 neonatal hospitals in Emilia Romagna region. 13.624 participants were followed up until their first birthday or season end. Among 292 infants hospitalized with RSV LRTI (2.1%), fewer were in the post- Nirsevimab than pre-Nirsevimab season group (72 [24.7%] vs. 220 [75.3%]; *p* < 0.001). Prematurity (HR, 2.93; 95%CI, 2.11–4.07; *p* < 0.001) and living with older siblings (HR, 4.57; 95%CI, 4.15–5.03; *p* < 0.001) remained associated with higher hospitalization risk among infants who received prophylaxis. Among hospitalized infants, Nirsevimab was associated with reduced HFNC use (OR, 0.33; 95%CI, 0.11–0.97; *p* = 0.04) but not with shorter stays (incidence rate ratio, 0.81; 95%CI, 0.63–1.03; *p* = 0.09). In conclusion Nirsevimab prophylaxis was associated with substantially lower RSV hospitalization risk and reduced in-hospital RSV severity [19].

In a systematic review and meta-analysis of a total of 50 publications, covering approximately 7.6 million people, Nirsevimab showed 80.7% effectiveness against RSV-related emergency department visits, 80.7% against hospital admissions and 75.6% against intensive care unit admissions [20]. In another systematic review and meta-analysis of 27 cohort and case-control studies Nirsevimab was highly effective in preventing RSV-related outcomes in infants, with a pooled real-world effectiveness of 83% against hospitalization, 81% against ICU admission, and 75% against LRTI [21].

A case–control study in a pediatric emergency department in Paris, that included infants younger than 12 months, has demonstrated that during the first French national immunization campaign, a single dose of Nirsevimab had an effectiveness of 83% against pediatric emergency department visits for RSV-associated bronchiolitis, 59% against hospitalizations for all-cause bronchiolitis, 83% against hospitalizations for RSV-associated bronchiolitis and 91% against the need oxygen supplement [22]. Another case-control study, conducted in a primary care setting with 107 ambulatory pediatricians, including all infants aged < 12 months, with bronchiolitis who performed an RSV rapid antigen test. The results show that 13.7% of case patients and 41.2% control patients were previously immunized for Nirsevimab. The adjusted effectiveness against RSV-bronchiolitis was estimated at 79.7%, confirming the benefit of immunization in primary care [23]. Furthermore, a large retrospective cohort study conducted in Catalonia (Spain) between October 2023 and January 2024, using a comprehensive database linking primary and hospital care data, reported an effectiveness of 87.6% against hospital admissions and 90.1% against ICU admissions for RSV-bronchiolitis in infants younger than six months. Importantly, this study also documented substantial reductions in less severe outcomes, such as all-cause bronchiolitis managed in primary care (48.1%), RSV infections (68.9%), and viral pneumonia (60.7%) [24].

In line with these findings, our data indicate that Nirsevimab reduced the risk of all-cause bronchiolitis in the outpatient pediatric setting by approximately 50%, after excluding premature infants. Specifically, bronchiolitis occurred in 8.8% (18/205) of not immunized infants and 6.5% (40/613) of immunized ones. Considering the entire population, 7.1% (58/818) of enrolled infants developed bronchiolitis after the start of the immunization campaign. When compared with internal data from the previous year (personal communication of a subgroup of the authors), where 20.7% of infants (41/198) developed bronchiolitis, a lower proportion of cases was observed in the 2024–2025 season (7.1% overall; 8.8% among non-immunized infants, 18/205). These comparisons are purely descriptive and should be interpreted cautiously. Although it is known from literature that a certain variability of RSV epidemiology is possible across years [25].

Our results also show that 74.9% of eligible children were immunized, a coverage rate comparable to the 76.3% reported in the Catalonian cohort [24]. Nevertheless, this figure emphasizes the need to strengthen both organizational aspects of immunization programs and communication strategies with parents, in order to maximize uptake.

Finally, our analysis shows that hospitalization for all -cause bronchiolitis was required in 2.4% (5/205) of not immunized infants and 1.8% (11/613) of immunized infants, respectively. In line with existing studies [22], our study suggests that Nirsevimab determines a 49.1% reduction in hospitalizations for all-cause bronchiolitis in the group of immunized children.

When we compare our results with those reported by Kampmann et al. on the efficacy of the bivalent RSV prefusion F protein–based (RSVpreF) vaccine in preventing medically attended RSV-associated lower respiratory tract illness (vaccine efficacy of 57.1% 90 days after birth and 51.3% 180 days after birth), the efficacy results are similar although some considerations should be made: first our study is a real world efficacy study and not a double-blind trial and moreover we included all the bronchiolitis diagnosis and not only the RSV associated form as an outcome [26].

Despite these encouraging results, our study also highlights some limitations. First, the immunization campaign in Lazio began on December 9, 2024, when the RSV season was already at its peak, with high transmission rates. Forty bronchiolitis cases occurred between November 1 and the campaign starting date and were excluded from the analysis. Considering the observed 50% risk reduction, we estimate that about 20 of these early cases could have been prevented if immunization had started earlier.

The delayed introduction of Nirsevimab in the Lazio region unfortunately impacted also on the bronchiolitis hospitalization rate. A recent study conducted in our region showed that the overall reduction of admissions, since introduction of Nirsevimab, did not consequently meet the expectations with only a 43% decrease in RSV bronchiolitis cases [27].

Second, bronchiolitis was diagnosed clinically without systematic use of RSV rapid antigen testing. As a result, our analysis included all-cause bronchiolitis, which reflects real-world pediatric practice but does not allow us to precisely assess effectiveness against RSV-specific bronchiolitis. This makes it difficult to compare our outcomes with those of the studies of other Italian regions and countries, where effectiveness of Nirsevimab against bronchiolitis prevalence and hospitalization is usually evaluated in RSV-related cases.

Third, the relatively small number of bronchiolitis events in our cohort may increase the sensitivity of effect estimates to minor changes in case counts, which could partially explain the difference between crude and IPW-adjusted estimates.

Fourth, it is important to highlight that using “all-causes bronchiolitis” as a parameter, may introduce potential bias for outcome misclassification. Bronchiolitis is caused by multiple viruses, and the incidence of the disease can be influenced over the years by changes in viral epidemiology, co-infection patterns, or secular seasonal trends, regardless of Nirsevimab. Consequently, the observed reduction in “all-cause bronchiolitis” cannot be attributed solely to protection against RSV-associated disease.

Future research should build on these findings by adopting more rigorous study designs to further clarify the real-world effectiveness of Nirsevimab. In particular, further prospective studies involving a larger number of primary care paediatricians (PPCs), with clearly defined and synchronized follow-up periods and standardized, laboratory-confirmed RSV outcomes, would allow for a more precise assessment of RSV-specific protection. A multi-season observation would be especially valuable to compare effectiveness estimates across different epidemic contexts and to account for interannual variability in RSV circulation. Expanding the range of measured confounders, incorporating formal power calculations, and performing sensitivity analyses would further strengthen causal inference. Together, these methodological enhancements would complement the present findings and contribute to a more definitive evaluation of Nirsevimab’s population-level impact in routine clinical practice.

In conclusion, our study provides evidence that Nirsevimab significantly reduces the risk of bronchiolitis in a real-world primary care setting. Our estimated effect is aligned with other reports although it could be underestimated due to the delayed immunization campaign and the inclusion of all-cause bronchiolitis. Taking together, our findings support the crucial role of Nirsevimab in preventing bronchiolitis, underscore the importance of early and widespread immunization, and highlight the need to optimize coverage and prioritize vulnerable groups in future campaigns.

## Data Availability

Deidentified data may be made available on reasonable request to the corresponding author.
